# Assessing the Role of Adalimumab in Treating Hidradenitis Suppurativa: Findings from a Retrospective Study at a Reference Center

**DOI:** 10.3390/clinpract14050135

**Published:** 2024-08-27

**Authors:** Austėja Šakaitytė, Inga Česnavičiūtė, Tadas Raudonis

**Affiliations:** 1 Clinic of Infectious Diseases and Dermatovenereology, Institute of Clinical Medicine, Faculty of Medicine, Vilnius University, 03101 Vilnius, Lithuania; inga.cesnaviciute@mf.vu.lt (I.Č.); tadas.raudonis@santa.lt (T.R.); 2 European Hidradenitis Suppurativa Foundation e.V., 06847 Dessau, Germany

**Keywords:** hidradenitis suppurativa, adalimumab, biological therapy, treatment outcome

## Abstract

Background: Hidradenitis suppurativa (HS) is a chronic inflammatory skin condition characterized by inflammatory lesions, often leading to scarring. Managing HS can be difficult, requiring biological therapy, specifically adalimumab. Methods: A retrospective study was conducted on patients diagnosed with HS and treated with the TNF-α inhibitor adalimumab. Data from 21 patients were included in this study. International Hidradenitis Suppurativa Severity Score System (IHS4); Dermatology Life Quality Index (DLQI); pain intensity according to the Visual Analogue Scale (VAS); and number of nodules, abscesses, and fistulas were assessed. Results: Notably, 47.62% of patients achieved Hidradenitis Suppurativa Clinical Response. The mean number of inflamed nodules decreased from 5.62 ± 4.12 to 3 ± 3.46, abscesses decreased from 1.76 ± 2.63 to 0.81 ± 1.4, and fistulas decreased from 2.62 ± 1.86 to 2 ± 1.9 (*p* < 0.05). The IHS4 score decreased from 19 ± 10.78 to 12.62 ± 11.13 (*p* = 0.001), DLQI from 15.76 ± 7.73 to 7.43 ± 7.76 (*p* < 0.001), and VAS from 6.69 ± 1.56 to 3.64 ± 2.65 (*p* < 0.001). There was a significant difference in the baseline IHS4 scores between patients who had prior surgery with a mean score of 23.86 ± 9.4 versus non-surgical patients with a mean IHS4 score of 9.29 ± 5.53 (*p* = 0.001). Conclusions: About half of HS patients responded positively to adalimumab treatment; the use of the drug reduces inflammatory lesions, and pain, and improves quality of life.

## 1. Introduction

Hidradenitis suppurativa (HS), also identified as acne inversa, is a persistent and recurrent inflammatory skin disease, marked by the development of deep, painful lesions including nodules, abscesses, fistulas, and subsequent scarring [1]. The condition predominantly appears in areas abundant with apocrine glands, such as the axilla, groin, anogenital, and inframammary areas [2]. The prevalence of HS varies among geographical regions, with the estimated range falling between 0.00033% and 4.1% [3]. In Europe and the United States, the occurrence of HS is roughly two times higher in females than in males, whereas in the Asia–Pacific region, a higher prevalence is observed in males [4]. The peak incidence of the condition is observed in individuals aged 20–40, with a decline in prevalence among those over 50. While the initial symptoms of the disease commonly manifest during the twenties, it can also impact pediatric patients, potentially due to a familial history of the condition [5,6]. Moreover, individuals experience symptoms for an average of 7.2 years before receiving a proper diagnosis of HS [7]. A delay in diagnosis results in irreversible scarring and has a significant impact on the patient’s physical and mental health, as well as their ability to participate in everyday work and social interactions [8]. Research suggests that a delay in initiating therapy is associated with a reduced response to adalimumab [9]. Managing HS can be challenging, requiring patients to undergo multiple extended treatment regimens of systemic antibiotics. In cases of mild HS (Hurley stage I–II), topical antibiotics, particularly 1% clindamycin, are the first-line treatment [10]. If there are numerous lesions and frequent flare-ups, systemic tetracyclines may be considered for treatment [11]. In patients with Hurley stage II/III and multiple active lesions, systemic clindamycin and rifampicin (at a dosage of 300 mg twice daily) for a typical duration of 10 weeks are recommended [12,13]. Guidelines for HS suggest that biologic therapy should be considered as an alternative if conventional treatments prove ineffective [14]. Specifically, the monoclonal antibody adalimumab, designed to target tumor necrosis factor (TNF)-α, demonstrated effectiveness compared to a placebo in two phase III trials, and for several years was the only biologic drug approved by the Food and Drug Administration (FDA) for the treatment of HS [15]. The response rate for this treatment reaches about 60% of patients based on the Hidradenitis Suppurativa Clinical Response (HiSCR), defined as a 50% reduction in the count of abscesses and inflammatory nodules with no increase in abscesses or draining fistula count; however, extensive long-term data from routine clinical practice for HS are still lacking [15,16,17]. Secukinumab, an anti-interleukin 17A medication, has recently been approved for adults with active moderate-to-severe HS who have not responded adequately to conventional HS therapy [18]. In the SUNSHINE and SUNRISE trials, the rates of achieving HiSCR with secukinumab ranged from 42% to 46%, compared to 31% to 34% with the placebo [19]. Infliximab, a biologic TNF-α inhibitor administered intravenously, has also demonstrated efficacy and may be a viable option for individuals with moderate-to-severe HS [20]. In a clinical trial, 26% of patients in the treatment group experienced a 50% or greater improvement, compared to only 5% in the placebo group; however, there is a lack of prospective studies regarding infliximab therapy [20,21]. Although new treatment methods have emerged, adalimumab remains the preferred biological agent in treatment algorithms, making it essential to evaluate its effectiveness [14].

Our study aimed to evaluate the efficiency of adalimumab in clinical practice among patients treated for moderate-to-severe HS at the Vilnius University Hospital Santaros Klinikos (VUH SK) Center of Dermatovenerology in Lithuania.

## 2. Materials and Methods

A retrospective study was carried out at the VUH SK Centre of Dermatovenereology after obtaining approval from the Vilnius Regional Biomedical Research Ethics Committee No. 2021/2˗1310˗793. All subjects were informed about the study and provided signed consent forms. The object of the study was patients who visited VUH SK Centre of Dermatovenereology, were diagnosed with HS, and were treated with adalimumab from 2018 to the end of 2023. Patients with moderate-to-severe HS who did not respond to previous systemic antibiotic therapy were included in our study. Data were collected from 23 patients; however, data from 2 patients were missing or could not be fully analyzed. Therefore, this study analyzed the medical histories of 21 patients. The anonymized dataset consisted of patients’ gender; age; height; weight; BMI; disease duration; Hurley stage; antibiotic and surgical treatment before adalimumab administration; IHS4; DLQI; VAS pain score; and number of nodules, abscesses, and fistulas, which were assessed both before and during treatment with adalimumab. This study has certain limitations, such as a small sample size, an unequal distribution of women and men, and an increase in the maintenance dose from 40 mg to 80 mg weekly in some patients.

Statistical data analysis was performed using Microsoft Excel 2021 and IMB SPSS 26. Descriptive statistics were employed to evaluate the prevalence of study characteristics within the sample. For qualitative (nominal) variables, frequency and relative frequency (percentages) were computed. The mean and standard deviation (SD) were calculated for quantitative variables. The normality of the distribution was assessed using the Shapiro–Wilk test. When data followed a normal distribution, the paired-sample *t*-test was utilized for two paired observations of quantitative data, while the non-parametric Wilcoxon test was employed for non-normally distributed data. The Mann–Whitney U test was used for quantitative parameters when there were two independent study groups. For quantitative parameters with more than two independent study groups, the Kruskal–Wallis test was used. Pearson chi-square and Fisher’s exact tests were used to analyze qualitative parameters. The results were considered significant if their *p*-values were below 0.05. 

## 3. Results

### 3.1. Demographics

In total, 21 patients diagnosed with moderate-to-severe HS were included in this study, comprising 8 women (38.1%) and 13 men (61.9%) with a mean age of 42.9 ± 14.1 years. The mean body mass index (BMI) of the patients was 30.33 ± 7.13, with eight patients (38.1%) considered overweight and eight (38.1%) considered obese (Table 1).

### 3.2. Clinical Characteristics

The majority of patients, constituting 13 individuals (61.9%), were classified as Hurley stage III. The mean duration of the disease was 15.48 ± 12.83 years. All 21 (100%) patients received prior systemic antibiotic treatment, 14 of whom (66.7%) had prior surgical treatment. The average baseline IHS4 score was 19 ± 10.78, with the majority of patients categorized as having severe HS. The initial DLQI score averaged 15.76 ± 7.73, signifying a very large effect of the disease on an individual’s quality of life. The mean pain intensity according to the VAS at baseline was 6.69 ± 1.59, indicating a moderate level of pain experienced by patients (Table 2).

### 3.3. Adalimumab Effectiveness

**Figure 1:**
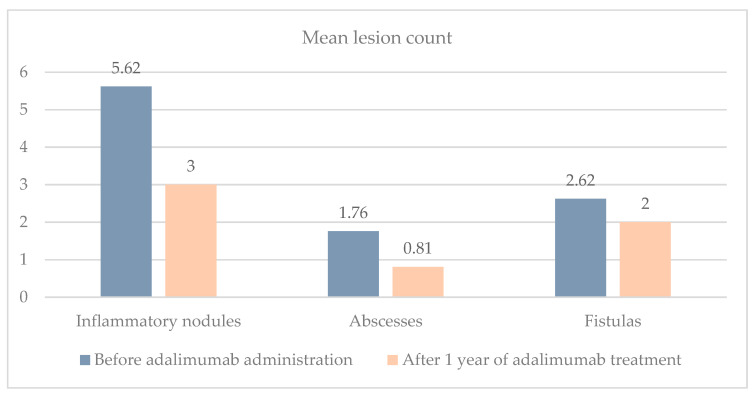
Mean lesion count of inflammatory nodules, abscesses, and fistulas before adalimumab administration and after 1 year of treatment.

Notably, 10 (47.62%) patients achieved HiSCR after one year of adalimumab treatment. A significant decrease in all HS lesions compared to the baseline counts was observed after one year of therapy (*p* < 0.05). The average count of inflammatory nodules reduced from 5.62 ± 4.12 to 3 ± 3.46, the mean count of abscesses decreased from 1.76 ± 2.63 to 0.81 ± 1.4, and the mean count of fistulas decreased from 2.62 ± 1.86 to 2 ± 1.9 (Figure 1).

**Figure 2:**
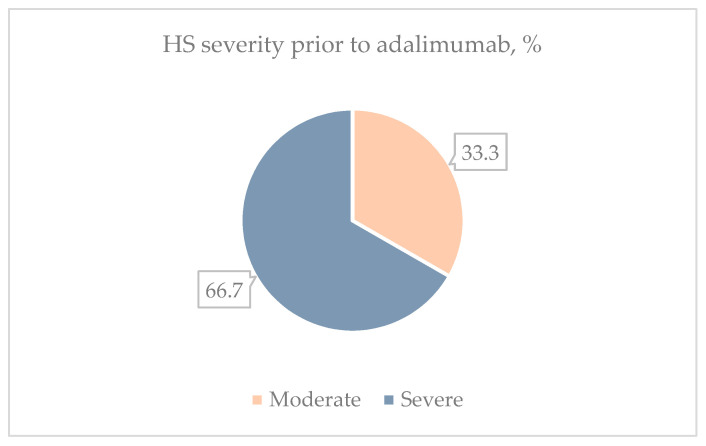

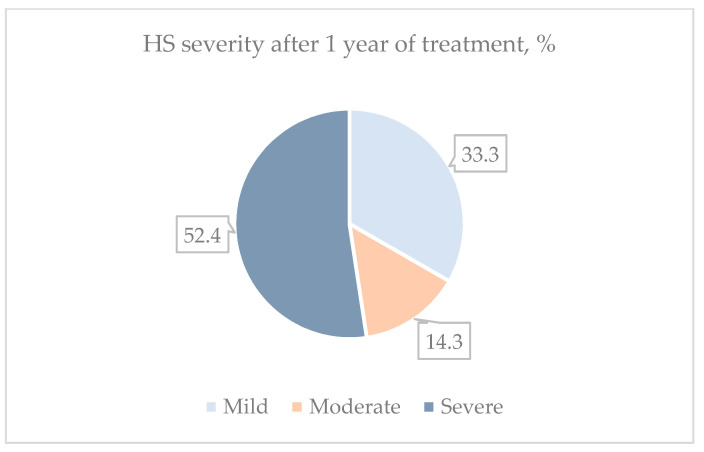
Distribution of HS severity categories before adalimumab treatment and after 1 year of treatment.

Before adalimumab administration, 14 patients (66.7%) had a severe HS according to the IHS4 scores, which decreased to 11 patients (52.4%) after treatment. Notably, there were no patients with mild HS initially, but after one year of treatment, seven patients (33.3%) with a moderate-to-severe form transitioned to the mild HS category (Figure 2).

**Figure 3:**
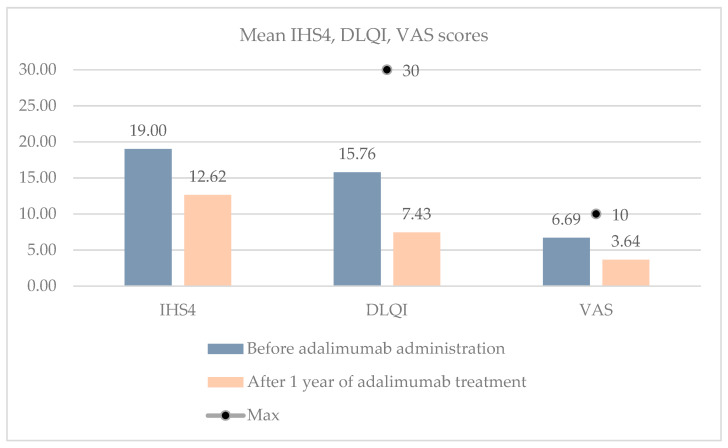
Mean IHS4, DLQI, and VAS scores before the administration of adalimumab and following one year of treatment.

A reduction in the number of inflammatory lesions corresponded with the IHS4 scores. The reduction in the IHS4 score after 1 year of adalimumab treatment was statistically significant compared to the baseline (*p* = 0.001). A mean IHS4 score of 19 ± 10.78 dropped to 12.62 ± 11.13. There was also a statistically significant enhancement in patients’ quality of life, as evidenced by the DLQI questionnaire (*p* < 0.001). At the baseline, the mean DLQI score was 15.76 ± 7.73, which decreased to 7.43 ± 7.76 after one year of treatment, indicating that on average, the very substantial effect on patients’ quality of life was reduced to moderate. Moreover, we assessed the VAS scores before initiating adalimumab treatment and after one year of therapy. The initial mean VAS score of 6.69 ± 1.59 decreased statistically significantly to 3.64 ± 2.65 (*p* < 0.001) (Figure 3).

### 3.4. Influence of BMI and Surgical Treatment

There was no significant association between BMI category (normal, overweight, and obese) and achievement of HiSCR (*p* = 0.350) (Table 3). The mean BMI of those who achieved the HiSCR was 32.17 ± 8.66, and those who did not achieve this clinical score had a mean BMI of 28.65 ± 5.27.

There were no statistically significant differences observed in the pre-treatment IHS4 estimate (*p* = 0.928), the baseline DLQI score (*p* = 0.232), or the baseline VAS score (*p* = 0.316) between patients with a normal BMI and those who are overweight or obese. The highest mean IHS4 and VAS values were in the group of patients with normal BMI (20.4 ± 13.01 and 7.4 ± 1.08, respectively); however, the highest mean DLQI (19.25 ± 7.23) was in the group of overweight patients (Table 4).

There was also no difference in the proportion of patients achieving a HiSCR between those who received prior surgical treatment and those who did not (*p* = 0.659) (Table 5).

There was a significant difference (*p* = 0.001) in the IHS4 scores before adalimumab treatment, with a mean score of 23.86 ± 9.4 for patients who had prior surgery, compared to the mean score of 9.29 ± 5.53 for those who had not undergone prior surgery. There were no statistically significant differences in the initial DLQI (*p* = 0.585) and VAS scores (*p* = 0.4) between patient groups with and without prior surgery for HS, with the mean scores being higher in patients who did not have prior surgery (Table 6).

## 4. Discussion

Mild HS cases are frequently treated with topical medications, while more advanced instances may necessitate systemic interventions such as oral antibiotics, retinoids, and immunosuppressants [22]. Despite the availability of therapeutic choices, managing HS remains challenging due to the considerable number of cases that do not respond to treatment [23]. The effectiveness of adalimumab in treating individuals with moderate-to-severe HS unresponsive to traditional treatments has been extensively established through two 12-week controlled clinical trials and two extension studies, suggesting its viability as a suitable option for medium-to-long-term management of HS [15,24,25]. HiSCR, a validated measure of outcome, is endorsed by robust evidence and is recommended for assessing the effectiveness of treatments in controlling inflammatory lesions in patients with HS [26].

Since adalimumab is prescribed for moderate-to-severe HS, patients typically have a long history of the disease and have often tried multiple treatments. In a multicenter observational study conducted in Japan, 39.8% of patients had HS for ≥10 years, and 84.3% of patients had previously received pharmacological treatment for HS before initiating adalimumab therapy [27]. A similar trend was observed in the PIONEER I and PIONEER II studies, where patients treated with adalimumab had an average history of HS lasting 8.8 and 9 years, respectively. Notably, 46.4% of patients in PIONEER I and 50.3% in PIONEER II received other systemic treatment prior to adalimumab [15]. In our study, patients had HS for an average of 15.48 years. In Lithuania, adalimumab is typically prescribed only after systemic antibiotic treatment has proven ineffective, so all patients had previously used antibiotics before starting adalimumab.

Our study shows the efficacy of adalimumab in clinical practice at the VUH SK Center of Dermatovenerology in Lithuania. A clinical response to adalimumab, evaluated by HiSCR after one year of treatment, was observed in 47.62% of patients, aligning with the results from previously documented controlled clinical trials [15,24,25]. These findings closely resemble those of a retrospective, real-life multicenter cohort study conducted in Italy, where 53.9% of patients attained HiSCR after one year (52 weeks) of treatment. 

Throughout adalimumab treatment, there was a significant decrease in the abscess, nodule, and fistula count. The outcomes align with the response observed in the study by Smetanova et al., where after 12 months of treatment, the reduction in mean values was as follows: Inflammatory nodules decreased from 8.15 to 2.83, abscesses from 2.98 to 0.67, and draining fistulas from 4.96 to 1.43 [28]. In our study, inflammatory nodules decreased from 5.62 to 3, abscesses from 1.76 to 0.81, and fistulas from 2.62 to 2.

The IHS4, often integrated as a secondary outcome measure, serves as a straightforward validation system adapted for routine clinical practice [29]. We also employed this scoring system to evaluate therapeutic responses within our study group. A mean IHS4 score dropped from 19 at the baseline to 12.62 after one year of treatment, and the proportion of severe HS decreased from 66.7% to 52.4%. A similar result was obtained by Chiricozzi et al., indicating that the mean IHS4 score after 52 weeks of adalimumab treatment decreased from 22.2 to 14.1, and the proportion of severe HS decreased from 85.4% to 50% [30].

The DLQI questionnaire and the VAS pain score are commonly recognized measurement tools for assessing the quality of life in HS patients [14,31,32]. In our study, there was a notable decrease in the baseline DLQI and VAS pain scores following one year of adalimumab treatment, with mean scores dropping from 15.76 to 7.43 and from 6.69 to 3.64, respectively. Smetanova et al. observed a similar trend, with the mean DLQI estimate decreasing from 17.61 to 6.08 [28], while Chiricozzi et al. reported a decrease in the mean VAS score from 5.5 to 2.7 [30].

A notable correlation between body weight and HS has been firmly established. The suggested pathophysiological mechanism linking obesity to HS involves heightened friction in flexural areas, a moist skin environment conducive to bacterial proliferation, and the systemic inflammation associated with elevated BMI [33,34]. A meta-analysis revealed that patients with HS had a 3.5 times greater probability of being obese than individuals in the control group [35]. In a retrospective cohort study of patients with HS, it was found that BMI was notably elevated compared to matched controls, with an average BMI among patients with HS being 31.51 kg/m^2^ [36]. In our study, the mean BMI of the patient was 30.33 kg/m^2^, and only 23.8% of them had a normal BMI. Although our study did not find a statistical association between BMI and the achieved clinical response (HiSCR), disease severity (IHS4), DLQI, and VAS, other studies suggest that higher BMI is linked to a more severe disease course and a poorer response to adalimumab treatment [37,38]. Weight loss and bariatric procedures are linked with a notable decrease in HS severity; however, it may exacerbate symptoms if it leads to a substantial increase in skin folds, necessitating the excision of excess skin [39,40].

Surgery has been a key component of HS management for a considerable period and is commonly utilized in patients with severe Hurley stage III disease [41]. In our study, a surgical procedure was performed for most patients (66.7%), with those patients having a significantly higher mean IHS4 score than patients who did not have surgery, at 23.86 and 9.29, respectively. This suggests that surgical treatment was more frequently administered to patients with a more severe form of the disease. Due to frequent recurrences of the disease, there has been a growing interest in utilizing targeted biological therapy for managing HS [42,43]. The study by Shanmugam et al. demonstrated that biologic therapy is linked to a quicker reduction in disease activity, particularly notable in patients who also underwent surgery for HS [44]. Aarts et al. study found that the combined approach of adalimumab with surgery resulted in notably greater clinical effectiveness and enhanced quality of life compared to adalimumab monotherapy, accompanied by increased patient satisfaction. Following 12 months of treatment, the surgery group demonstrated a significantly greater decrease in IHS4 scores than the monotherapy group (mean reduction of −9.1 versus −7.8). Moreover, the surgery group experienced a more substantial decrease in DLQI scores after treatment compared to the monotherapy group (mean reduction of −8.2 versus −4) [45]. In our study, no significant difference was observed in the achieved clinical response (HiSCR) between individuals who received surgical treatment before adalimumab administration and those who did not; however, we did not investigate the effect of surgical treatment during adalimumab treatment.

## 5. Conclusions

Approximately half of the 21 patients achieved HiSCR with adalimumab treatment for HS. Adalimumab effectively decreased the number of inflammatory lesions such as nodules, abscesses, and fistulas. Throughout the treatment course, there was a notable decrease in disease severity and pain levels, and an enhancement in quality of life. No significant association was found between the BMI category and the achievement of HiSCR. Patients who underwent prior surgery had significantly higher IHS4 scores before adalimumab treatment compared to those who did not undergo prior surgery.

## Figures and Tables

**Table 1:** Patient demographics characteristics.

Characteristic	Patients (*n* = 21)
Sex	
Female, *n*, (%)	8 (38.1%)
Male, *n*, (%)	13 (61.9%)
Mean age, years, (±SD)	42.9 (±14.1)
Mean BMI kg/m^2^ (±SD)	30.33 (±7.13)
Normal (18.5–24.9), *n*, (%)	5 (23.8%)
Overweight (25.0–29.9), *n*, (%)	8 (38.1%)
Obese (≥30.0), *n*, (%)	8 (38.1%)

**Table 2:** Baseline clinical characteristics.

Characteristic	Patients (*n* = 21)
Hurley stage, *n*, (%)	
II	8 (38.1%)
III	13 (61.9%)
Mean duration of HS, years, (±SD)	15.48 (±12.83)
Prior systemic antibiotic use, *n*, (%)	21 (100%)
Prior surgery for HS, *n*, (%)	14 (66.7%)
Lesion counts	
Mean no. of inflammatory nodules (±SD)	5.62 (±4.12)
Mean no. of abscesses (±SD)	1.76 (±2.63)
Mean no. of fistulas (±SD)	2.62 (±1.86)
Mean IHS4 score (±SD)	19 (±10.78)
Moderate (4–10), *n*, (%)	7 (33.3%)
Severe (≥11), *n*, (%)	14 (66.7%)
Mean DLQI score (±SD)	15.76 (±7.73)
Mean VAS score (±SD)	6.69 (±1.59)

**Table 3:** Association between BMI category and achievement of HiSCR.

	Achieved HiSCR (*n* = 10)	Did Not Achieve HiSCR (*n* = 11)	Total (*n* = 21)	*p*-Value
Normal BMI, Count (%)	3 (60%)	2 (40%)	5	0.350
Overweight, Count (%)	5 (62.5%)	3 (37.5%)	8	
Obese, Count (%)	2 (25%)	6 (75%)	8	

**Table 4:** Distribution of initial mean IHS4, DLQI, and VAS estimates between BMI groups.

	Normal BMI	Overweight	Obese	*p*-Value
Mean IHS4 (±SD)	20.4 (±13.01)	19.75 (±13.18)	17.38 (±7.52)	0.928
Mean DLQI (±SD)	12.8 (±7.95)	19.25 (±7.23)	14.13 (±7.68)	0.232
Mean VAS (±SD)	7.4 (±1.08)	6.13 (±1.71)	6.81 (±1.69)	0.316

**Table 5:** Association between patients with or without prior surgical treatment and the attainment of HiSCR.

	Achieved HiSCR (*n* = 10)	Did Not Achieve HiSCR (*n* = 11)	Total (*n* = 21)	*p*-Value
Had prior surgical treatment, count (%)	6 (42.86%)	8 (57.14%)	14	0.659
Did not have prior surgery, count (%)	4 (57.14%)	3 (42.86%)	7	

**Table 6:** Distribution of initial mean IHS4, DLQI, and VAS estimates between patients who had surgical treatment and those who did not.

	Prior Surgery	No Prior Surgery	*p*-Value
Mean IHS4 (±SD)	23.86 (±9.4)	9.29 (±5.53)	0.001
Mean DLQI (±SD)	15.5 (±8.92)	16.29 (±5.12)	0.585
Mean VAS (±SD)	6.46 (±1.82)	7.14 (±0.9)	0.4

## Data Availability

The data supporting the findings of this study can be obtained from the corresponding author upon reasonable request.
